# Immunomodulatory effect of IFN-γ licensed adipose-mesenchymal stromal cells in an in vitro model of inflammation generated by SARS-CoV-2 antigens

**DOI:** 10.1038/s41598-024-75776-5

**Published:** 2024-10-16

**Authors:** Elizabete Cristina Iseke Bispo, Enrique Roberto Argañaraz, Franscisco de Assis Rocha Neves, Juliana Lott de Carvalho, Felipe Saldanha-Araujo

**Affiliations:** 1 Laboratory of Hematology and Stem Cells (LHCT), Faculty of Health Sciences, University of Brasília, Brasília, 70910-900 Brazil; 2 Laboratory of Molecular NeuroVirology, Faculty of Health Sciences, University of Brasília, Brasília, 70910-900 Brazil; 3 Laboratory of Molecular Pharmacology, Faculty of Health Sciences, University of Brasília, Brasília, 70910-900 Brazil; 4 Interdisciplinary Laboratory of Bioscience, Faculty of Medicine, University of Brasília, Brasília, 70910-900 Brazil

**Keywords:** Mesenchymal stem cells, IFN-γ, T-cells, Nucleocapsid, Spike, SARS-CoV-2, COVID-19, Biotechnology, Drug discovery, Stem cells

## Abstract

In recent years, clinical studies have shown positive results of the application of Mesenchymal Stromal Cells (MSCs) in severe cases of COVID-19. However, the mechanisms of immunomodulation of IFN-γ licensed MSCs in SARS-CoV-2 infection are only partially understood. In this study, we first tested the effect of IFN-γ licensing in the MSC immunomodulatory profile. Then, we established an in vitro model of inflammation by exposing Calu-3 lung cells to SARS-CoV-2 nucleocapsid and spike (NS) antigens, and determined the toxicity of SARS-CoV-2 NS antigen and/or IFN-γ stimulation to Calu-3. The conditioned medium (iCM) generated by Calu-3 cells exposed to IFN-γ and SARS-CoV-2 NS antigens was used to stimulate T-cells, which were then co-cultured with IFN-γ-licensed MSCs. The exposure to IFN-γ and SARS-CoV-2 NS antigens compromised the viability of Calu-3 cells and induced the expression of the inflammatory mediators *ICAM-1*,* CXCL-10*, and *IFN-β* by these cells. Importantly, despite initially stimulating T-cell activation, IFN-γ-licensed MSCs dramatically reduced IL-6 and IL-10 levels secreted by T-cells exposed to NS antigens and iCM. Moreover, IFN-γ-licensed MSCs were able to significantly inhibit T-cell apoptosis induced by SARS-CoV-2 NS antigens. Taken together, our data show that, in addition to reducing the level of critical cytokines in COVID-19, IFN-γ-licensed MSCs protect T-cells from SARS-CoV-2 antigen-induced apoptosis. Such observations suggest that MSCs may contribute to COVID-19 management by preventing the lymphopenia and immunodeficiency observed in critical cases of the disease.

**Supplementary Information:**

The online version contains supplementary material available at 10.1038/s41598-024-75776-5.

## Introduction

Mesenchymal Stromal Cells (MSCs) are described as multipotent progenitor cells, with fibroblastoid morphology and the ability to differentiate into different types of cells such as adipocytes, osteoblasts, and chondrocytes^1^. MSCs can be obtained from various adult and fetal tissues, including the bone marrow, cord blood, dental pulp, adipose tissue, liver, lung, and placenta, among others^2–4^. Regarding their functional properties, it has been demonstrated that fetal MSCs tend to show greater proliferative capacity, differentiation, and fitness, compared to adult MSCs^5,6^.

Importantly, MSCs have been receiving special attention due to their capacity to interact with the immune system^7^, which makes them interesting for clinical use^8,9^. Although the mechanisms underlying the immunomodulatory function of MSCs are not completely understood, they appear to involve a complex network, that involves the inhibition of T-cell activation and the generation of regulatory T-cells^10^ through cell-cell contact^11^ and the secretion of various immunomodulatory molecules, including adenosine^12^, transforming growth factor-β (TGF-β)^13^, indoleamine 2,3-dioxygenase (IDO)^14^, IL-10^15^, and TNF-α-stimulated gene 6 protein (TSG-6)^16^, among others. Interestingly, it has been demonstrated that following infusion, MSCs undergo apoptosis in the presence of cytotoxic cells and that this is a requirement for their immunosuppressive function^17^.

Interestingly, it has been demonstrated that the licensing of MSCs with cytokines, other biomolecules, and chemical agents, increases their potential to secrete factors that regulate signaling pathways associated with inflammatory response, tissue repair and angiogenesis^18^. Licensing MSCs with IFN-γ is one of the most investigated strategies to enhance the immunosuppressive potential of these cells^19,20^. In addition to enhancing the anti-inflammatory phenotype of these cells, this licensing strategy ensures a protective effect of MSCs against cryopreservation^21^. However, despite the use of licensed MSCs being seen as a new generation of MSC therapy, reports of clinical use of such licensed cells are still extremely scarce in the literature.

Potentially, licensed MSCs could be used for a series of pathologies in which unlicensed MSCs are already being applied, including Graft-versus-Host Disease (GvHD)^22^, diabetes^23^, rheumatoid arthritis^24^, Systemic Lupus Erythematosus (SLE)^25^, Crohn’s disease^26^, and COVID-19^27^. Nevertheless, MSC-licensing has not been experimentally tested for most of these applications.

Severe Acute Respiratory Syndrome Coronavirus 2 (SARS-CoV-2), the causative agent of Coronavirus Disease 2019 (COVID-19), has been responsible for a worldwide pandemic causing high rates of morbidity and mortality (https://covid19.who.int/*).* This virus has structural proteins that include spike (S), envelope (E), membrane (M), and nucleocapsid (N)^28^. Importantly, the N protein is abundantly expressed during infection caused by SARS-CoV-2 and, together with the S protein, represents the main immunogens of COVID-19^29,30^.

The SARS-CoV-2 infection initiates by interaction between Spike-S1 subunit protein receptor binding domain (RBD) with the Angiotensin Converting Enzyme 2 (ACE2). Then, the transmembrane serine protease 2 (TMPRSS2) and the disintegrin and metalloprotease 17 (ADAM17) promote the cleavage of the S2 subunit and viral and host cell membrane fusion^31,32^. After cell entry, the virus genome and viral proteins are recognized by pattern recognition receptors (PRRs) that start a signaling cascade, inducing interferon (IFN) I and III production^33^ along with pro-inflammatory cytokines and chemokines^34,35^. Both T-cells and monocyte macrophages migrate from the peripheral blood to the site of infection and stimulate the production of more cytokines, leading to the inflammatory response^31,36^. When the immune system is unable to resolve the infection, an imbalance in the release of cytokines may occur, leading to a hyperactive state, known as cytokine storm. Such an exacerbated release of pro-inflammatory cytokines can cause systemic inflammation and excessive tissue damage, leading to multiple organ failure^37^.

Although clinical studies show positive results from the application of MSCs in severe cases of COVID-19, the immunomodulation mechanisms involved in this scenario are only partially established at this point. There is also a lack of data in the literature regarding the potential of IFN-γ-licensed MSCs to control the inflammation generated by SARS-CoV-2. In this study, we established an in vitro model of inflammation by exposing Calu-3 lung cells to the immunogenic SARS-CV-2 N and S proteins (NS) and tested the ability of IFN-γ-licensed MSCs to modulate T-cells response in this environment.

## Materials and methods

### MSCs culture and characterization

MSCs were obtained from three healthy donors following liposuction procedure^38,39^. These cells were cultured using alpha - Minimum Essential Medium (α-MEM) supplemented with 15% fetal bovine serum (FBS – Gibco, USA), 2 mM glutamine, and 100 U/mL penicillin/streptomycin (Sigma, USA), at 37 °C and 5% CO_2_. The media was changed every 2 days until 70–90% confluency. MSCs between passages 3–6 were used for all experiments. The study protocols were approved by the Ethics Committee of the Faculty of Health Sciences of the University of Brasilia. All methods described in the study were carried out in accordance with the approved guidelines. All participants provided written informed consent.

MSCs were phenotypically characterized by flow cytometry (FACSCalibur, BD Bioscience, USA) using the BD Stemflow™ hMSC Analysis kit (Pharmingen, BD Biosciences, USA) following manufacturer’s instructions. In brief, this kit contains antibodies to evaluate the expression of CD44, CD73, CD90, and CD105. Additionally, the kit has a negative cocktail, which includes antibodies raised against CD45, CD34, CD11b, CD19 and HLA-DR. To determine the immunophenotype of MSCs, cells from the 3rd passage were harvested and stained. A total of 10,000 events were acquired from each sample. Data were analyzed using FlowJo Software 10.0.7 (FlowJo LLC, USA, https://www.flowjo.com/). MSCs from 3rd to 6th passages were used for all experiments.

### MSC licensing

MSCs were cultured for 48 h with 25 ng/mL, 50 ng/mL, or 100 ng/mL IFN-γ (GIBCO, EUA). After this period, the culture medium was discarded and the cells were washed 3x with PBS to completely remove the IFN-γ. Then, the cells were tested for their ability to control the proliferation of T-cells.

## Immunosuppression assay

For the immunosuppression assay, PBMCs were obtained from healthy volunteers using Histopaque 1077 (Sigma-Aldrich, USA). After counting, the cells were stained with 2.5 µM carboxylfluorescein succinimidyl ester (CFSE) (ThermoFisher, USA) allowing proliferation analysis. The labeled PBMCs were stimulated with 5 µg/mL of phytohaemagglutinin (PHA, Sigma-Aldrich, USA) and cocultured with IFN-γ licensed MSCs at a 10 PBMCs to 1 MSC (10:1) ratio for 120 h. After this period, PBMCs were collected, stained with anti-CD3 APC (BD Pharmingen, USA), and T-cell proliferation was determined by flow cytometry after the collection of 10,000 events. The analysis was performed using FlowJo Software 10.0.7 (FlowJo LLC, USA, https://www.flowjo.com/).

## MTT assay in Calu-3 cells exposed to NS antigens

To assess the proliferation/viability of Calu-3 cells (purchased from ATCC) exposed to NS antigens, we performed the 3-(4.5-dimethylthiazol-2-yl)-2,5-diphenyl tetrazolium bromide (MTT, Sigma-Aldrich, USA) assay in presence of an inflammatory *milieu* generated by the use of SARS-CoV-2 Nucleoprotein and Spike protein antigens and IFN-γ. For this, a total of 5 × 10^3^ Calu-3 cells were seeded in a 96 well plate and, after 24 h, cells were exposed to 50 ng/mL IFN-γ (GIBCO, USA) and 2 µg/mL NS antigens (SARS-CoV-2 Nucleoprotein/Spike Protein (N-RBD), Thermo Fisher, USA). After 48 h of culture, 10 µl of MTT (5 mg/mL) was added to each well and the plate was incubated for 4 h. Then, the media was discarded and DMSO was added to dissolve the reaction product. The absorbance was read using a Multiskan FC Microplate Photometer (Thermo Scientific, USA) at 570 nm.

## Determination of Lactate dehydrogenase (LDH) release

We assessed the LDH release by Calu-3 cells treated with 50 ng/mL IFN-γ and 2 µg/mL NS antigens using the CytoTox 96 kit, according to manufacturer’s instructions (Promega, USA). Briefly, cells were plated in a 12-well plate and exposed to 50 ng/mL IFN-γ and 2 µg/mL NS antigens. After 48 h, 100 µl of the supernatant were transferred to a 96-well plate and 50 µl of CytoTox 96 Reagent were added to each well. Then, the reaction was stopped and the absorbance was measured in a DTX 800 Multimode Detector spectrophotometer (Beckman Coulter, USA) at 492 nm.

## Caspase-1 inflammasome assay

Caspase-1 activity was determined in Calu-3 cells that were treated or not with 50 ng/mL IFN-γ and 2 µg/mL NS antigens, using the Caspase-Glo^®^1 inflammasome assay kit (Promega Corp., Madison, WI, USA), in accordance with manufacturer’s instructions. Briefly, after 48 h of culture, 100 µL of the Caspase-Glo 1 reagent was added to the plate and mixed using a plate shaker at 300 rpm for 30 s. After incubating the samples for 60 min at room temperature, the luminescence was measured using a Multimode Plate Reader (PerkinElmer, Waltham, MA, USA). To ensure the specificity of caspase-1 activity, reactions were also performed in parallel with the caspase-1 inhibitor Ac-YVAD-CHO, which does not inhibit cross-reactive caspases. The results of caspase 1 activity were normalized, subtracting the reading of samples without Ac-YVAD-CHO from the reading of samples treated with the caspase 1 inhibitor.

### Calu-3 conditioned medium

**Fig. 1:**
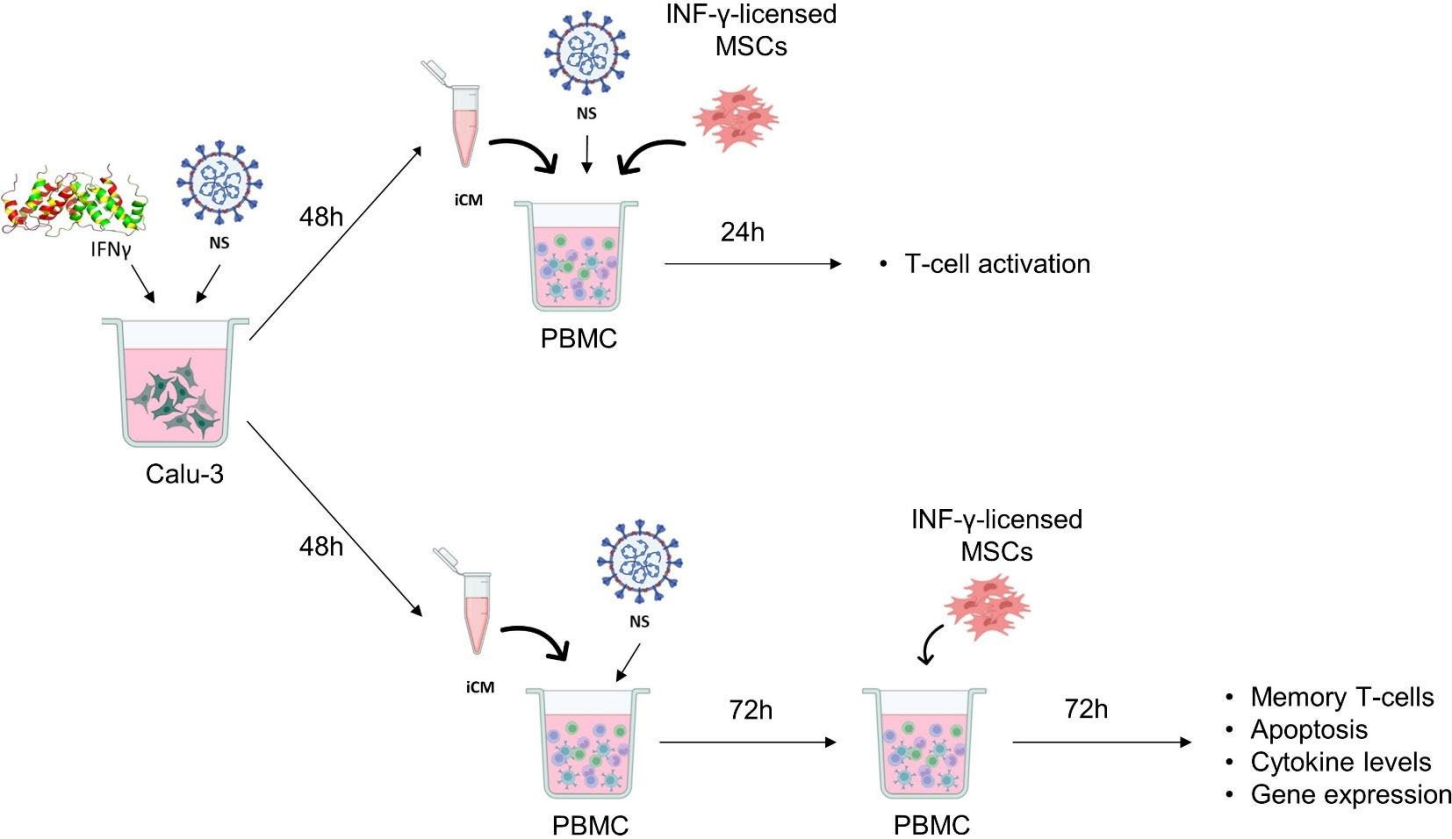
Experimental design. Calu-3 lung cells were exposed to IFN-γ and SARS-CoV-2 NS antigens. After 48 h, this inflamed conditioned medium (iCM) was collected and used in the proposed experiments. PBMCs were exposed to NS antigens in iCM medium and treated for 24 h with IFN-γ-licensed MSCs. After this period, the expression of activation markers was determined in T-cells. In parallel, PBMCs were exposed to NS antigens in iCM medium and after 72 h they were treated with IFN-γ-licensed MSCs. Following 72 h of treatment, the percentage of memory T-cells, the production of pro- and anti-inflammatory cytokines, the levels of T-cell apoptosis, and the expression of genes related to the immune response and cell death in both MSCs and PBMCs were determined.

Calu-3 cells were treated for 48 h with DMEM containing 2 µg/mL NS antigens and 50 ng/mL IFN-γ (iCM). Then, the inflammatory medium produced by Calu-3 (iCM) was used to stimulate PBMCs in different experimental conditions (Fig. 1).

## PBMCs obtention and T-cell activation assay

To assess T-cell activation, we isolated PBMCs from 5 donors that had COVID-19 and were vaccinated against SARS-CoV-2, using histopaque 1077. PBMCs were maintained in iCM (100 µl per well) and stimulated with 1 µg/mL NS antigens. The PBMCs were cultured alone or in the presence of IFN-γ licensed MSCs (10:1 ratio). After 24 h in such conditions, PBMCs were collected and stained with the following-conjugated antibodies: CD69-FITC (Invitrogen), CD137-PE (Invitrogen), CD3-PerCP (Invitrogen), CD3-FITC (BD Pharmingen), CD38-APC (Invitrogen), and CD25-PerCP (Invitrogen). A total of 10,000 events were recorded for each sample and data were analyzed using FlowJo Software 10.0.7 (FlowJo LLC, USA, https://www.flowjo.com/).

## Memory T-cell quantification

For quantification of memory T cells, isolated PBMCs were cultured in RPMI medium and exposed or not to 1 µg/mL NS antigens for 72 h in iCM. Then, IFN-γ-licensed MSCs were added to the PBMCs exposed to NS antigens and iCM (10:1 ratio) and cocultured for 72 h. After this period, the PBMCs were collected and stained with the following-conjugated antibodies: CD3-PE (Invitrogen), CD197 (CCR7)-FITC (Invitrogen) and CD45RA-APC (Invitrogen). A total of 30,000 events were recorded for each sample and data were analyzed using FlowJo Software 10.0.7 (FlowJo LLC, USA, https://www.flowjo.com/). For data analysis, the percentage of naïve T-cells (CD3+, CD45RA + and CCR7+), central memory T-cells (TCM) (CD3+, CD45RA- and CCR7+), effector memory T-cells (TEM) (CD3+, CD45RA- and CCR7-), and terminally differentiated memory effector T-cells (TEMRA) (CD3+, CD45RA + and CCR7-)^40^ were determined.

### Apoptosis detection

To determine the percentage of apoptotic CD4 and CD8 T-cells, PBMCs were exposed to 1 µg/mL NS antigens and cultured in iCM for 72 h. After this period, IFN-γ licensed MSCs (10:1 ratio) were added to the well and cocultured for 72 h. Then, cells were collected and stained with anti-CD4-APC, anti-CD8-PE, and Annexin V-FITC (Invitrogen), following manufacturer’s instructions. A total of 10,000 events were recorded for each sample and data were analyzed using FlowJo Software 10.0.7 (FlowJo LLC, USA, https://www.flowjo.com/).

### Real -time qPCR

Gene expression analysis was performed on MSCs and Calu-3 cells. For RNA extraction from MSCs that were co-cultured with PBMCs, a transwell system with a membrane pore size of 0.4 μm (Greiner Bio-one) was used to ensure that there would be no PBMC contamination in the RNA obtained. RNA extraction was performed using the TRI Reagent (Sigma-Aldrich, USA), according to the manufacturer’s recommendations. Samples were quantified using Nanodrop One (ThermoFisher, USA). Total RNA was reverse-transcribed using the High-Capacity cDNA Reverse Transcription Kit (Applied BioSystem, USA), and quantitative PCR was performed using the GoTaq qPCR Master Mix and GoTaq Probe qPCR Master Mix (Promega Corp., USA). The genes evaluated were *ACTB*, *ANGPT1*, *ANGPT2*, *BAX*, *CASP-1*,* CASP-8*,* CXCL10*,* EGF*, *FGF-2*, *GSDMD*, *HGF*, *ICAM-1*,* IFN-β*,* IFN- γ*,* IL-1β*,* IL-6*,* IL-10*,* IDO*, *PDL-1*,* P53*,* TGF-β*,* TGS-6*, *TNF-α*, and *VEGF* (Supplementary Table 1). *ACE2* (Hs01085333_m1) and *TMPRSS2* (Hs01122322_m1) transcriptional levels were assessed in Calu-3 and A549 cell lines using TaqMan probes. The reactions were performed in technical duplicates, and the relative fold change was obtained by the 2^−ΔΔCt^ method^41^. The mean Ct values were obtained from samples using control IFN-γ licensed MSCs or Calu-3 cells as a reference.

### Cytometric bead array

The amounts of IL-2, IL-6, IL-10, TNF-α and IFN-γ were determined in cell culture supernatants, using the CBA Human Th1/Th2 Cytokine Kit II, according to the instructions from the manufacturer (BD Bioscience, USA). For this, PBMCs were exposed to 1 µg/mL NS antigens and cultured for 72 h in iCM. Then, IFN-γ licensed MSCs (10:1 ratio) were added to the well and cocultured for 72 h. After this period, the supernatants were collected, assayed on a LSR Fortessa flow cytometer (BD Biosciences) and analyzed with the CBA Analysis Software (BD Bioscience, USA). Concentration of cytokines in samples was calculated using individual standard curves and expressed as pg/ml.

### Statistical analysis

Student’s t-test was used when comparing two groups, and ANOVA was used in comparing three groups. Probability values of *p* < 0.05 were accepted as indications of statistically significant differences and significance levels were defined as * *p* < 0.05; ** *p* < 0.01; *** *p* < 0.001; and **** *p* < 0.0001. All analyses were performed using Prism 9 software (GraphPad Software Inc., San Diego, CA, USA, https://www.graphpad.com/). Data were reported as mean ± SEM.

## Results

### MSCs characterization

**Fig. 2:**
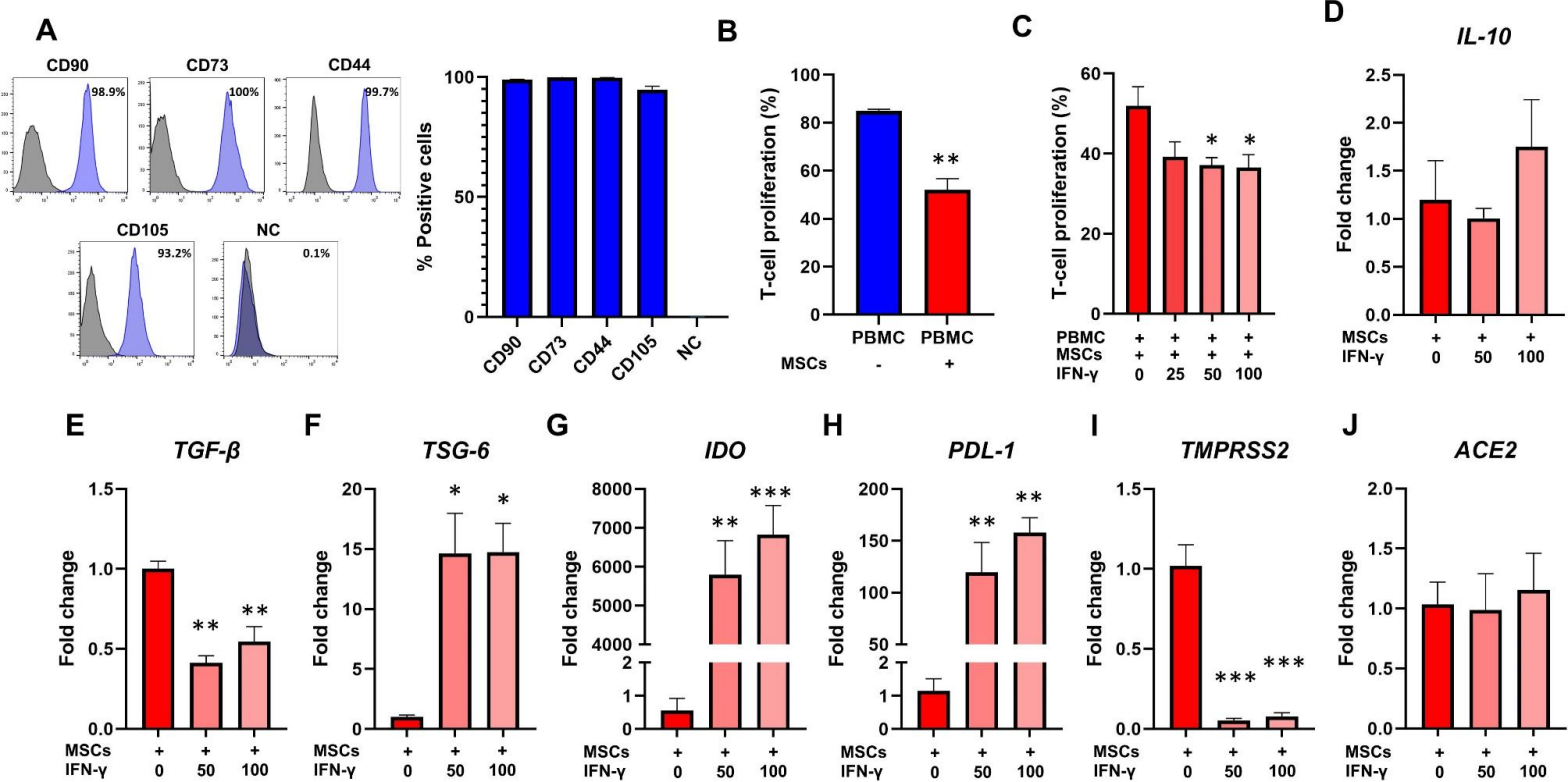
Characterization of MSCs. (A) Expression levels of CD90, CD73, CD44, CD105, and negative cocktail (NC) antibodies on MSCs. Representative flow cytometry histograms are shown on the left. (B) Proliferation of T-cells after their activation with PHA and co-culture with MSCs (10:1 ratio). (C) Proliferation of T-cells after their activation with PHA and co-culture with control MSCs or MSCs previously licensed with increasing doses of IFN-γ. (D-J) Gene expression analysis of *IL-10*, *TGF-β*, *TSG-6*, *IDO*, *PDL-1*, *TMPRSS2*, and *ACE2* in MSCs or IFN-γ-licensed MSCs. The fold changes were determined by the 2^− ΔΔCt^ method, using the mean Ct value of MSCs as a reference. Results are presented as mean ± SEM. *p < 0.05; **p < 0.01; ***p < 0.001.

As expected, the immunophenotypic profile of MSCs showed CD44+, CD73+, CD90+, CD105+, CD34-, CD45-, CD11b-, CD19-, and HLA-DR- (Fig. 2A).

### Licensing with IFN-γ increased the immunosuppressive capacity of MSCs

MSCs significantly controlled the proliferation of T-cells (*p* = 0.001) (Fig. 2B). More importantly, licensing MSCs with 50 ng/ml and 100 ng/ml significantly increased their ability to control T-cell division (*p* = 0.04 and *p* = 0.03, respectively, compared to unlicensed cells) (Fig. 2C). Licensing with 50 ng/ml and 100 ng/ml of IFN-γ did not alter *IL-10* transcriptional levels and caused a reduction in *TGF-β* expression in MSCs (*p* = 0.001 and *p* = 0.006, respectively) (Fig. 2D, E). On the other hand, licensing with 50 ng/ml and 100 ng/ml of IFN-γ significantly increased the expression of *TSG-6* (*p* = 0.01), *IDO* (*p* = 0.002 and *p* = 0.0008, respectively), and *PD-L1* (*p* = 0.009 and *p* = 0.002, respectively) in MSCs (Fig. 2F-H), compared to the unlicensed counterparts. Interestingly, licensing with 50 ng/ml and 100 ng/ml of IFN-γ also reduced *TMPRSS2* transcriptional levels in MSCs (*p* = 0.0003). No changes were seen with respect to *ACE2* mRNA levels (Fig. 2I, J).

### NS antigens induce the death of Calu-3 cells in an inflammatory environment

**Fig. 3:**
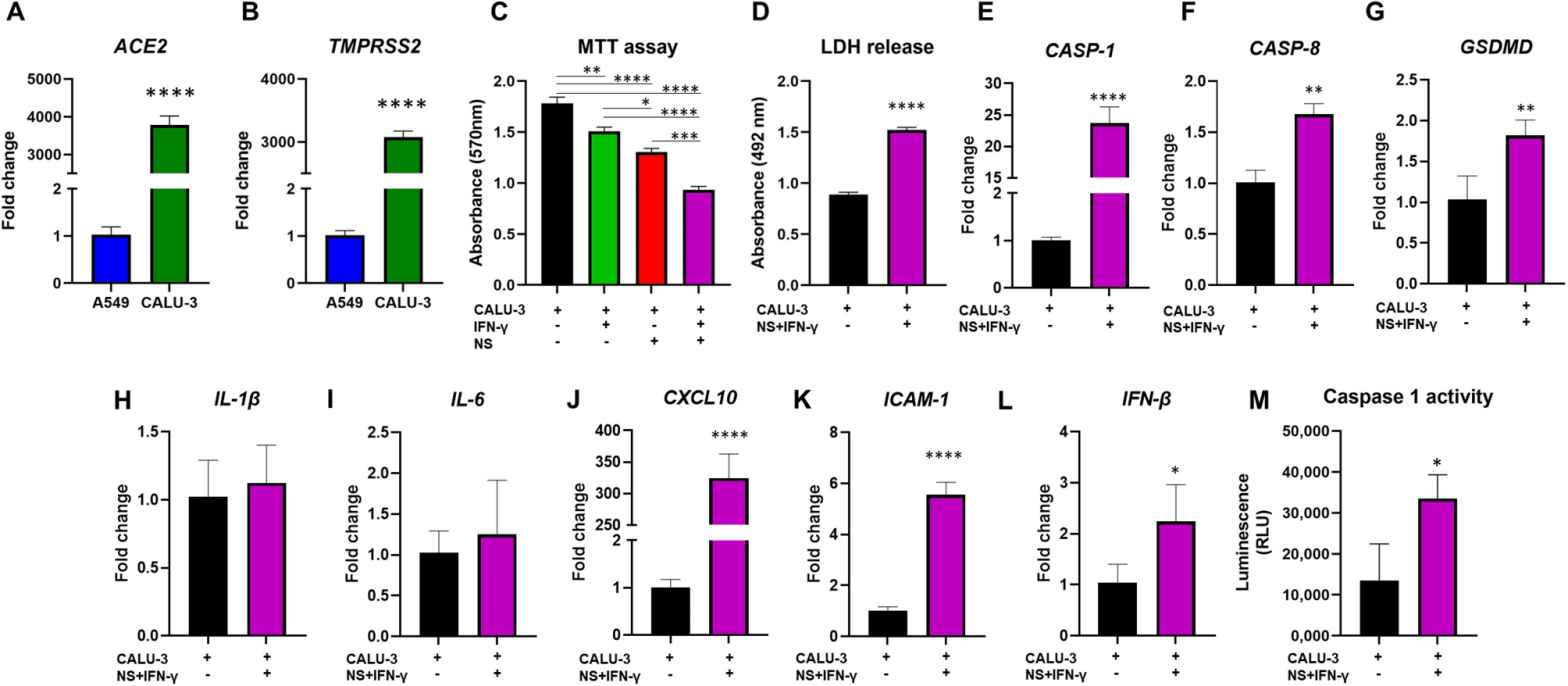
Characterization of the viability and inflammatory status of Calu-3 cells exposed to NS antigens. (A) *ACE2* and *TMPRSS2* expression in Calu-3 and A549 lung cell lines. The fold changes were determined by the 2^− ΔΔCt^ method, using the mean Ct value of A549 as a reference. (C) Cell viability determined by MTT of Calu-3 cells exposed to IFN-γ and NS antigens. (D) LDH release by Calu-3 exposed to IFN-γ and NS antigens. (E-L) Transcriptional levels of *CASP-1*, *CASP-8*, *GSDMD*, *IL-1β*, *IL-6*, *CXCL10*, *ICAM-1*, and *IFN-β* in Calu-3 cells exposed or not to IFN-γ and NS antigens. The fold changes were determined by the 2^− ΔΔCt^ method, using the mean Ct value of Calu-3 cells as a reference. (M) Caspase 1 activity in Calu-3 cells exposed or not to IFN-γ and NS antigens. Results are presented as mean ± SEM. *p < 0.05; **p < 0.01; ***p < 0.001; ****p < 0.0001.

Initially, we evaluated the transcriptional levels of SARS-CoV-2 entry receptors in two pulmonary epithelial cell lines, Calu-3 and A549. Calu-3 cells were chosen for the following experiments due to the greater mRNA levels of *ACE2* (*p* < 0.0001) and *TMPRSS2* (*p* < 0.0001), compared with A549 cells (Fig. 3A, B). Then, we investigated the viability of Calu-3 cells in the presence of NS antigens and IFN-γ. Interestingly, 50 ng/ml IFN-γ was able to impair the viability of Calu-3 cells (*p* = 0.004). These cells also showed reduced viability when exposed to NS antigens (*p* < 0.0001). However, the greatest toxicity to Calu-3 cells was caused by the exposure of these cells to the NS antigen in an inflammatory environment induced by the presence of IFN-γ (*p* < 0.0001) (Fig. 3C). The deleterious effect of IFN-γ and NS antigen on Calu-3 cells was validated by LDH measurement. In agreement with the MTT assay, the supernatant obtained from the culture of Calu-3 cells with IFN-γ and NS antigen showed high levels of LDH, compared to the supernatant from unexposed Calu-3 cells (*p* < 0.0001) (Fig. 3D). After characterizing the toxicity promoted by inflammation and the NS antigen on lung cells, we evaluated the expression of transcripts related to death and inflammation in these cells. Calu-3 cells exposed to IFN-γ and NS had an increase in the transcriptional levels of *CASP-1* (*p* < 0.0001), *CASP-8* (*p* = 0.001), and *GSDMD* (*p* = 0.008), compared to unexposed cells (Fig. 3E-G).

Calu-3 cells exposed to IFN-γ and NS antigens did not show modulation in the transcriptional levels of *IL-1β* and *IL-6* (Fig. 3H, I). On the other hand, these cells showed increased expression of *CXCL10* (*p* < 0.0001), *ICAM-1* (*p* < 0.0001), and *IFN- β* (*p* = 0.03) (Fig. 3J-L).

### Caspase 1 activity in Calu-3 cells

As expected, Calu-3 cells exposed to IFN-γ and NS antigens had greater caspase 1 activity than control Calu-3 cells (*p* = 0.01) (Fig. 3M).

### IFN-γ-licensed MSCs induce T-cell activation after coculture with PBMCs exposed to NS antigens

**Fig. 4:**
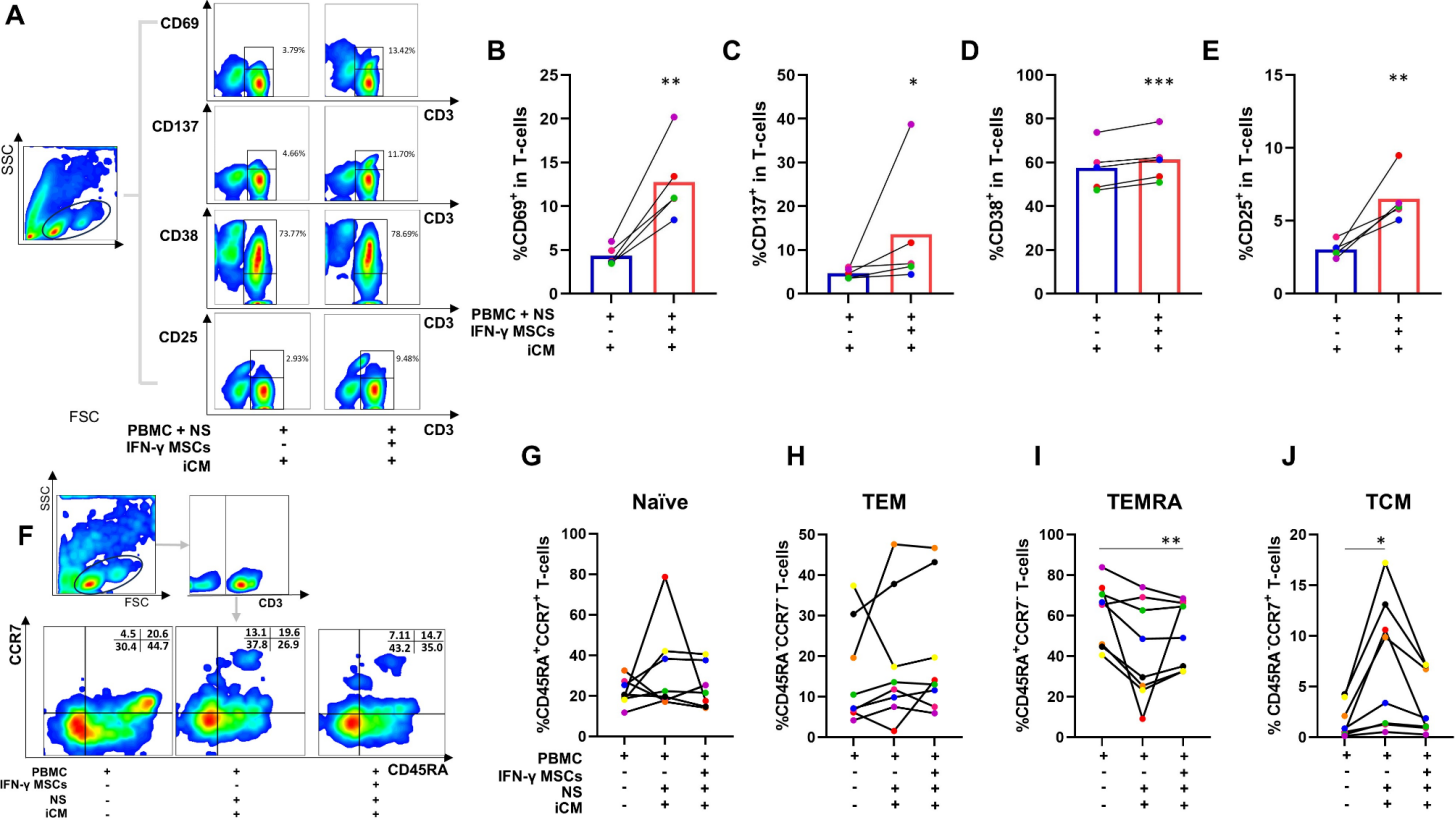
Assessment of activation markers and memory T-cells. (A-E) Expression of CD69, CD137, CD38, and CD25 on T-cells after the culture of PBMCs with NS antigens in iCM, and coculture or not with IFN-γ-licensed MSCs. Representative flow cytometry plots of T-cells positive for CD69, CD137, CD38, and CD25 are shown on the left. (F-J) Percentage of naive T-cells, TEM, TEMRA, and TCM after culture of PBMCs in RPMI medium, with NS antigens in iCM, and coculture with IFN-γ-licensed MSCs. Representative gating strategy and flow cytometry plots of CCR7 and CD45RA expression on T-cells are shown on the left. *p < 0.05; **p < 0.01; ***p < 0.001.

The effects of IFN-γ-licensed MSCs on the expression of T-cell activation markers CD69, CD137, CD38 and CD25 were determined by flow cytometry in PBMCs maintained in iCM, cocultured with MSCs, and exposed to NS antigens. Interestingly, IFN-γ-licensed MSCs induced increased expression of activation markers CD69 (*p* = 0.003), CD137 (*p* = 0.03), CD38 (*p* = 0.0006) and CD25 (*p* = 0.007) in T-cells, after culturing PBMCs in iCM (Fig. 4A-E).

### IFN-γ-licensed MSCs decreases the percentage of memory T-cells in PBMCs exposed to NS antigens

PBMCs exposed to NS antigens in iCM medium showed an increase in TCM cells (*p* = 0.03). Although not statistically significant, the presence of MSCs reduced the percentage of TCM cells by 31% (average). The presence of IFN-γ-licensed MSCs did not alter the percentage of naïve T cells and TEM, after exposure of PBMCs to NS antigens in iCM. On the other hand, MSCs induced a significantly lower percentage of TEMRA cells (*p* = 0.006), compared to PBMCs that were isolated and cultured in RPMI medium (Fig. 4F-J).

### IFN-γ-licensed MSCs protect PBMCs from apoptosis induced by NS antigens

**Fig. 5:**
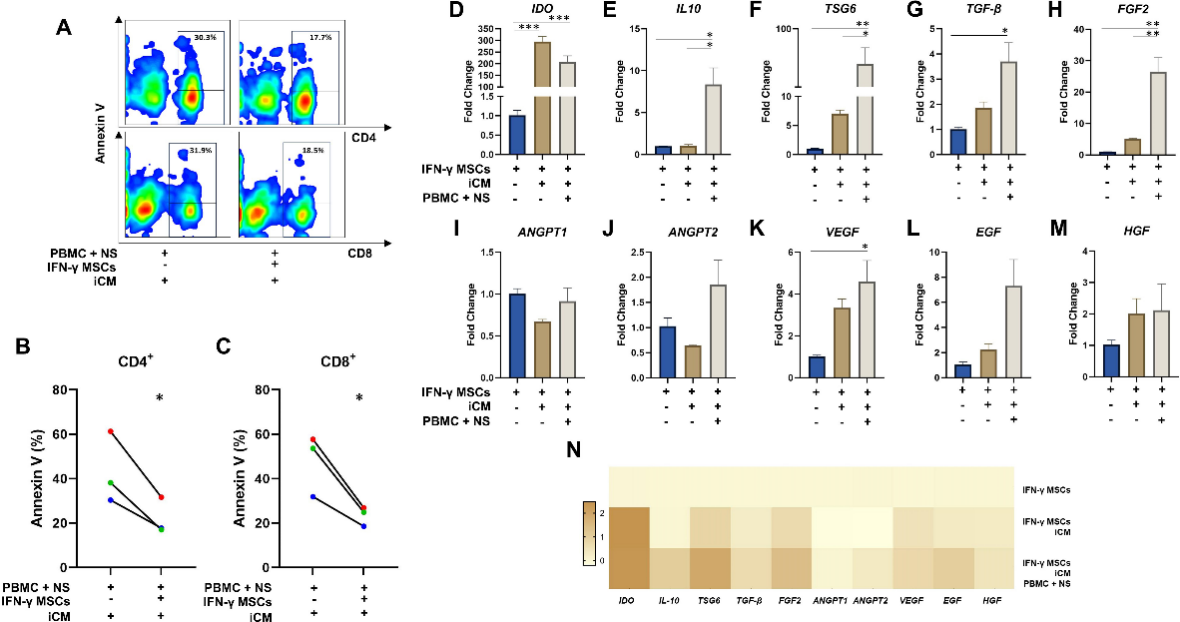
T-cell apoptosis and gene expression analysis. (A) Representative flow cytometry plots of annexin-V staining on CD4 and CD8 T-cells. (B, C) Percentage of annexin-V + CD4 and CD8 T-cells after the culture of PBMCs with NS antigens in iCM, and treatment or not with IFN-γ-licensed MSCs. (D-M) Transcriptional levels of *IDO*, *IL-10*, *TSG-6*, *TGF-β*, *FGF2*, *ANGPT1*, *ANGPT2*, *VEGF*, *EGF*, and *HGF* in IFN-γ licensed MSCs, IFN-γ-licensed MSCs that were cultured in iCM medium, and IFN-γ-licensed MSCs that were co-cultured with PBMCs in iCM medium. (N) Heatmap showing the expression of inflammatory, regenerative, and apoptosis-related genes in IFN-γ licensed MSCs, IFN-γ-licensed MSCs that were cultured in iCM medium, and IFN-γ-licensed MSCs that were co-cultured with PBMCs in iCM medium. All real-time PCRs reactions were performed in technical duplicate, and the relative fold change was obtained with the 2^−∆∆Ct^ method. Data are expressed as mean with SEM. *p < 0.05; **p < 0.01; ***p < 0.001.

Importantly, the presence of IFN-γ-licensed MSCs significantly reduced the levels of apoptosis of T-CD4 (*p* = 0.02) and T-CD8 cells (*p* = 0.02) after exposure of PBMCS to NS antigens in iCM, compared to PBMCs that were not cocultured with MSCs (Fig. 5A-C).

#### Expression of anti-inflammatory, regenerative and anti-apoptotic genes in MSCs after co-culture with PBMCs

IFN-γ-licensed MSCs that were co-cultured with PBMCs in iCM medium showed increased expression of genes with anti-inflammatory roles, such as *IL-10* (*p* = 0.02), and *TSG-6* (*p* = 0.01), compared with IFN-γ-licensed MSCs that were cultured in iCM. *IDO* expression was not altered between these two groups, although exposure of IFN-γ-licensed MSCs to iCM medium increased the levels of this transcript (*p* = 0.0001), as occurred in IFN-γ-licensed MSCs that were co-cultured with PBMCs in iCM medium (*p* = 0.0008), compared to IFN-γ-licensed MSCs not subjected to iCM nor NS antigens (Fig. 5D-F). *TGF-β* transcriptional levels were higher in IFN-γ-licensed MSCs that were co-cultured with PBMCs in iCM medium, compared to IFN-γ-licensed MSCs (*p* = 0.03) (Fig. 5G). Also, IFN-γ-licensed MSCs that were co-cultured with PBMCs in iCM medium showed increased expression *FGF2* (*p* = 0.01), compared with IFN-γ-licensed MSCs that were cultured in iCM medium (*p* = 0.008). No statistically significant changes were observed with respect to *ANGPT1* and *ANGPT2* transcriptional levels (Fig. 5H-J). *VEGF* transcriptional levels were higher in IFN-γ-licensed MSCs that were co-cultured with PBMCs in iCM medium, compared to IFN-γ-licensed MSCs (*p* = 0.04) (Fig. 5K). Although we did not identify statistically significant differences, *EGF* transcriptional levels increased by 4.6 fold in IFN-γ-licensed MSCs that were co-cultured with PBMCs in iCM medium, compared to IFN-γ-licensed MSCs that were cultured in iCM medium, and 10.1 fold compared to IFN-γ-licensed MSCs. No statistically significant changes were observed regarding *HGF* expression (Fig. 5L-N).

### IFN-γ-licensed MSCs modulate the production of inflammatory factors by PBMCs exposed to NS antigens

**Fig. 6:**
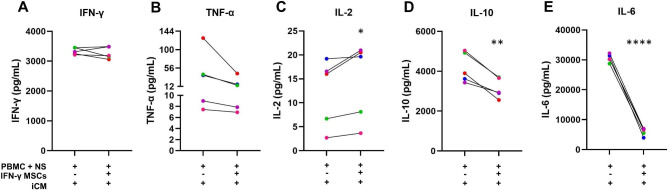
Cytokine quantification. Levels of (A) IFN-γ, (B) TNF-α, (C) IL-2, (D) IL-10, and (E) IL-6 secreted in the culture of PBMCs exposed to NS antigens in iCM, treated or not with IFN-γ-licensed MSCs. *p < 0.05; **p < 0.01; ****p < 0.0001.

The presence of IFN-γ-licensed MSCs did not significantly alter the levels of IFN-γ and TNF-α produced by PBMCs exposed to NS antigens in iCM (Fig. 6A, B). Interestingly, IFN-γ-licensed MSCs induced the production of IL-2 (*p* = 0.02) by PBMCs exposed to NS antigens in iCM (Fig. 6C). Furthermore, licensed cells significantly inhibited the production of IL-10 (*p* = 0.005) and IL-6 (*p* = 0.03) by PBMCs exposed to NS antigens in iCM (Fig. 6D, E).

## Discussion

The immunosuppressive and regenerative potential of MSCs served as a rationale for the development of a series of clinical studies that explored the use of these cells to treat COVID-19. Although there is great variability between studies, especially regarding the dose and source of MSCs, several authors show that the infusion of MSCs is safe and is associated with clinical improvement in patients with COVID-19^42^. In sharp contrast to the various clinical studies that have been conducted to treat COVID-19 with MSCs, information on the interaction of MSCs with lung and immune cells, and about the mechanisms by which MSCs can act against SARS-CoV-2 infection, is extremely scarce in the literature. Therefore, in the present study we investigated how IFN-γ-licensed MSCs influence T-cell response in a microenvironment generated by exposure of Calu-3 lung cells to SARS-CoV-2 NS antigens.

Our study was developed using MSCs obtained from adipose tissue, due to the numerous advantages of using these cells for regenerative medicine. In addition to their acquisition involving a minimally invasive liposuction procedure, the concentration of MSCs in adipose tissue tends to be higher than in other tissues within the body. Compared to bone marrow MSCs, adipose tissue-derived counterparts have a greater proliferative capacity, are more resistant to senescence, and are genetically more stable in prolonged cultures. Furthermore, there is evidence that adipose tissue MSCs regulate the immune response more efficiently than bone marrow MSCs^43,44^. Such aspects are supported by the fact that clinical trials conducted during the COVID-19 pandemic were largely based on MSCs from umbilical cord, adipose tissue and bone marrow^27,45^.

Compared to their unlicensed counterparts, IFN-γ-licensed MSCs showed increased expression of *IDO*, *PD-L1*,* TSG6*, as well as a greater efficiency in controlling the division of activated T-cells. Interestingly, it has been demonstrated that MSCs from bone marrow, umbilical cord, and adipose tissue express very low levels of ACE2 and TMPRSS2^46,47^. Importantly, our data show that IFN-γ licensing induces a reduction in *TMPRSS2* transcriptional levels in MSCs, which is particularly relevant and indicates that these cells may be even more resistant to SARS-CoV-2 infection.

SARS-CoV-2 virus infects lung cells by mediating membrane fusion through ACE2 and TMPRSS2; upon viral entry, an exacerbated immune response is initiated and culminates in damage to the lung tissue and dysfunction of multiple organs^48,49^. In our model, exposure to inflammation induced by IFN-γ and NS antigens compromised the viability of Calu-3 cells and induced greater production of inflammatory mediators, such as *ICAM-1*,* CXCL10* and *IFN-*β. Interestingly, the exposure of Calu-3 cells to IFN-γ and NS antigens modulated the expression of components involved in the inflammasome, such as *CASP1* and *GSDMD*. Together with the increased caspase 1 activity, these findings corroborate previous studies that showed the occurrence of pyroptosis in lung cells exposed to SARS-CoV-2 and its antigens^50,51^. Furthermore, we noticed increased levels of *CASP-8* in Calu-3 cells exposed to IFN-γ and NS antigens. Caspase-8 plays an important role in triggering both extrinsic and intrinsic pathways of apoptosis and is involved in SARS-CoV-2-induced lung cell apoptosis^52^. IFN-β is one of the main cytokines produced during SARS-CoV-2 infection and plays an important role in the spread of the virus, by stimulating the expression of ACE2 in lung endothelial cells^53^. In COVID-19, elevated levels of CXCL10 have been reported to be associated with a severe course and progression of the disease^54,55^. Furthermore, serum ICAM-1 levels are associated with lung injury^56^, being elevated in patients with mild disease, while dramatically elevated in severe cases^57^.

Considering that both lung cell injury and increased production of inflammatory mediators were detected in our experimental model, we used the supernatant produced by Calu-3 cells exposed to IFN-γ and NS antigens (iCM medium) as a microenvironment to evaluate the ability of IFN-γ-licensed MSCs to modulate T-cell responses. Interestingly, in this environment, MSCs were able to stimulate the expression of T-cell activation markers CD25, CD38, CD69, and CD137. CD25 and CD69 are well-established early markers of T-cell activation. CD69 expression can be rapidly stimulated after TCR/CD3 engagement, which triggers the release of inflammatory factors and T-cell proliferation^58^. Notably, MSCs are able to induce late CD69 expression on T-cells, which is associated with an immunosuppressive T-cell phenotype^59^. CD38 and CD137 expression is present on activated T- and B-cells. It has been shown that CD137 expression can enhance the immune response in acute viral infections^60^. Indeed, CD38 and CD137 expression is detected on SARS-CoV-2-reactive T cells^61^. Although the increase in the expression of activation markers induced by MSCs may seem controversial — considering their immunosuppressive role — it has been demonstrated that adipose tissue MSCs can induce the activation of T-cells, before controlling their function^62–64^.

Memory T-cells are heterogeneous cells that can be identified based on membrane markers. In particular, the expression of the surface molecules CD45RA and CCR7 allows the classification of human T-cells into naive T-cells, TCM, TEM, and TEMRA^65^. Interestingly, the treatment of PBMCs exposed to iCM with IFN-γ-licensed MSCs did not alter the percentage of naïve and TEM, but promoted a reduction in TEMRA cells. Interestingly, the reduction in the percentage of TEMRA cells has been observed both in convalescent patients, when compared to patients in the acute phase of COVID-19, and in patients with mild COVID, when compared to patients admitted to the intensive care unit^66,67^. Furthermore, we identified that the presence of MSCs reduced by 31% the percentage of TCM cells in PBMCs exposed to NS antigens in iCM medium. Although these results come from the exposure of PBMCs to viral antigens, the reduction in the percentage of TCM cells is an effect that raises concern, considering the important role of these cells in cases of reinfection by SARS-CoV-2.

We also evaluated transcriptional changes in IFN-γ-licensed MSCs after their contact with PBMCs and NS in iCM medium. Such cells showed increased expression of the anti-inflammatory transcript *IL-10*, compared with IFN-γ-licensed MSCs that were cultured in iCM. Interestingly, after contact with PBMCs in iCM, IFN-γ-licensed MSCs also presented increased expression of *TSG-6*. Importantly, in addition to being an anti-inflammatory mediator, TSG-6 has also been described as playing an important role in tissue protection^68^. Likewise, the investigated MSCs also showed increased expression of *TGF-β*, which — in addition to being involved in the immunomodulatory mechanisms of MSCs^69^ — constitutes an important antiapoptotic factor produced by MSCs^70^. FGF2 has an antifibrotic and protective role against lung injuries^71^, and its production by MSCs, as well as that of VEGF, appears to be associated with the regenerative role of MSCs in COVID-19^72^. Importantly, our data show that after exposure to PBMCs and NS in iCM, IFN-γ-licensed MSCs presented increased mRNA levels of both genes.

The excessive immune response present in SARS-CoV-2 infection is accompanied by the production of several cytokines, including IL-2, IFN-γ, TNFα, IL-10, and IL-6 ^73^. Interestingly, treatment of PBMCs exposed to NS antigens in iCM with IFN-γ-licensed MSCs caused an increase in IL-2 production, and a dramatic reduction in IL-10 and IL-6 levels. It is important to note that IFN-γ-licensed MSCs showed an increase in *IL-10* expression when in contact with PBMCs with NS in iCM, but in this environment the secreted levels of IL-10 were lower than the levels found in the isolated culture of PBMCs with NS in iCM. These findings are important, since elevated levels of IL-6 and IL-10 are found in critically ill COVID-19 patients and are associated with severe disease pattern^74–76^.

Lymphopenia is one of the main anomalies of the immune system of patients infected with SARS-CoV-2, contributing to the immunodeficiency, viral replication and death observed in severe cases of COVID-19^77^. Several factors are being associated with the lymphocyte reduction observed in COVID-19, with emphasis on the apoptotic process of T-cells triggered by the virus, and the compromised viability of immune cells caused by the exacerbated inflammation^78–80^. Importantly, in addition to controlling the levels of critical cytokines in COVID-19, our data shows that IFN-γ-licensed MSCs drastically reduced the apoptosis of CD4 and CD8 T-cells caused by the exposure of PBMCs to NS antigens. Interestingly, the presence of IFN-γ-licensed MSCs promoted an increase in IL-2 in the culture of PBMCs with NS in iCM and, even though high levels of IL-2 are associated with severe cases of COVID-19^81^, this cytokine plays an important role in T-cell survival^82^.

We emphasize that the results obtained in our study are derived from the interaction of IFN-γ-licensed MSCs and PBMCs in an environment containing the secretome of lung cells exposed to the NS antigen. In this context, the development of studies using MSCs, PBMCs and lung cells in spheroid models can greatly contribute to a better understanding of the effects resulting from the direct interaction between these cells in the presence of SARS-CoV-2 antigens. We also emphasize that an important limitation of our study is that our immunophenotypic analyses are based on the total T-cell population and do not discriminate between CD4 and CD8 T-cell subtypes. Furthermore, our study does not compare the immunomodulatory potential of IFN-γ-licensed MSCs and their unlicensed counterparts.

Taken together, our data show that IFN-γ-licensed MSCs are capable of modulating the immune response triggered by the inflammatory milieu produced by lung cells exposed to NS antigens, reducing the levels of critical cytokines in COVID-19, such as IL-10 and IL-6. Furthermore, in our model, IFN-γ-licensed MSCs were also able to exert an anti-apoptotic effect on T-cells, indicating that the use of MSCs can potentially contribute to avoiding lymphopenia and immunodeficiency observed in critical cases of COVID-19.

## Electronic supplementary material

Below is the link to the electronic supplementary material.

Supplementary Material 1

## Data Availability

Data will be made available from the corresponding author on reasonable request.
