# Supplementary material for: Immunomodulatory effect of IFN-γ licensed adipose-mesenchymal stromal cells in an in vitro model of inflammation generated by SARS-CoV-2 antigens
Source: Sci Rep. 2024 Oct 16;14:24235. doi: 10.1038/s41598-024-75776-5 (PMC11484699; doi:10.1038/s41598-024-75776-5)
Supplement: Supplementary file 1 — Supplementary Material 1 [file 41598_2024_75776_MOESM1_ESM.docx]

**Table1. qPCR primer sequences**

| **Gene** | **Primer** | **Sequence (5’- 3’)** |
| --- | --- | --- |
| *ACTB* | Forward | AGGCCAACCGCAAGAAG |
|  | Reverse | ACAGCCTGGATAGCAACGTACA |
| *ANGPT1* | Forward | CAATGGGGGAGGTTGGACTGTA |
|  | Reverse | GAGGGATTTCCAAAACCCATTTTAT |
| *ANGPT2* | Forward | ACGTGAGGATGGCAGCGTT |
|  | Reverse | GAAGGGTTACCAAATCCCACTTTAT |
| *BAX* | Forward | CAGACCGTGACCATCTTTGT |
|  | Reverse | GCCTCAGCCCATCTTCTTC |
| *CASP-1* | Forward | AAGACCCGAGCTTTGATTGACTC |
|  | Reverse | AAATCTCTGCCGACTTTTGTTTCC |
| *CASP-8* | Forward | GGATGGCCACTGTGAATAACTG |
|  | Reverse | TCGAGGACATCGCTCTCTCA |
| *CXCL10* | Forward | TGGCATTCAAGGAGTACCTC |
|  | Reverse | TTGTAGCAATGATCTCAACACG |
| *EGF* | Forward | CTTGTCATGCTGCTCCTCCTG |
|  | Reverse | TGCGACTCCTCACATCTCTGC |
| *FGF-2* | Forward | CTGGCTATGAAGGAAGATGGA |
|  | Reverse | TGCCCAGTTCGTTTCAGTG |
| *GSDMD* | Forward | ATGAGGTGCCTCCACAACTTCC |
|  | Reverse | CCAGTTCCTTGGAGATGGTCTC |
| *HGF* | Forward | CATGCTGGCCCTTACCTAGC |
|  | Reverse | GAGGAGAGGACCAAGTTCACA |
| *ICAM-1* | Forward | GGAGCTTCGTGTCCTGTATGGC |
|  | Reverse | CAGTGATGATGACAATCTCATACCG |
| *IFN-β* | Foward | GCTTGGATTCCTACAAAGAAGCA |
|  | Reverse | ATAGATGGTCAATGCGGCGTC |
| *IFN-γ* | Forward | ACTGTCGCCAGCAGCTAAAA |
|  | Reverse | TATTGCAGGCAGGACAACCA |
| *IL-1β* | Forward | AGAAGTACCTGAGCTCGCCA |
|  | Reverse | TGTTTAGGGCCATCAGCTTCA |
| *IL-6* | Forward | TCAATATTAGAGTCTCAACCCCCA |
|  | Reverse | TTCTCTTTCGTTCCCGGTGG |
| *IL-10* | Forward | GGCACCCAGTCTGAGAACAG |
|  | Reverse | ACTCTGCTGAAGGCATCTCG |
| *IDO* | Forward | GGGAAGCTTATGACGCCTGT |
|  | Reverse | CTGGCTTGCAGGAATCAGGA |
| *PD-L1* | Forward | AAACAATTAGACCTGGCTG |
|  | Reverse | TCTTACCACTCAGGACTTG |
| *TGF-β* | Forward | GCTGTATTTAAGGACACCGTGC |
|  | Reverse | TGACACAGAGATCCGCAGTC |
| *TSG-6* | Forward | CCCAGGTTGCTTGGCTGATT |
|  | Reverse | GGACCCATACGTACCTTCCC |
| *TNF-α* | Forward | CACAGTGAAGTGCTGGCAAC |
|  | Reverse | GATCAAAGCTGTAGGCCCCA |
| *VEGF* | Forward | GGGCAGAATCATCACGAAGT |
|  | Reverse | GTCTCGATTGGATGGCAGTAG |
